# An Eco-Friendly Process to Extract Hydroxyapatite from Sheep Bones for Regenerative Medicine: Structural, Morphologic and Electrical Studies

**DOI:** 10.3390/jfb14050279

**Published:** 2023-05-17

**Authors:** Sílvia Rodrigues Gavinho, Mehmet Bozdag, Cevriye Kalkandelen, Joana Soares Regadas, Suresh Kumar Jakka, Oguzhan Gunduz, Faik Nuzhet Oktar, Manuel Pedro Fernandes Graça

**Affiliations:** 1 I3N and Physics Department, University of Aveiro, 3810-193 Aveiro, Portugal; silviagavinho@ua.pt (S.R.G.); joanaregadas@live.ua.pt (J.S.R.); suresh@ua.pt (S.K.J.); 2 Department of Bioengineering, Faculty of Engineering, Goztepe Campus, Marmara University, 34722 Istanbul, Turkey; m.muratbozdag@hotmail.com (M.B.); foktar@marmara.edu.tr (F.N.O.); 3 Vocational School of Technical Sciences, Istanbul University-Cerrahpasa, 34722 Istanbul, Turkey; 4 Department of Metallurgy and Materials Engineering, Faculty of Technology, Goztepe Campus, Marmara University, 34722 Istanbul, Turkey; 5 Center for Nanotechnology & Biomaterials Applications and Research, Goztepe Campus, Marmara University, 34722 Istanbul, Turkey

**Keywords:** biomaterials, eco-friendly, natural hydroxyapatite, magnesium oxide, electrical properties, bone regeneration

## Abstract

Hydroxyapatite (HA) promotes excellent bone regeneration in bone-tissue engineering, due to its similarity to bone mineral and its ability to connect to living tissues. These factors promote the osteointegration process. This process can be enhanced by the presence of electrical charges, stored in the HA. Furthermore, several ions can be added to the HA structure to promote specific biological responses, such as magnesium ions. The main objective of this work was to extract hydroxyapatite from sheep femur bones and to study their structural and electrical properties by adding different amounts of magnesium oxide. The thermal and structural characterizations were performed using DTA, XRD, density, Raman spectroscopy and FTIR analysis. The morphology was studied using SEM, and the electrical measurements were registered as a function of frequency and temperature. Results show that: (i) an increase of MgO amount indicates that the solubility of MgO is below 5%wt for heat treatments at 600 °C; (ii) the rise of MgO content increases the capacity for electrical charge storage; (iii) sheep hydroxyapatite presents itself as a natural source of hydroxyapatite, environmentally sustainable and low cost, and promising for applications in regenerative medicine.

## 1. Introduction

As the most used material in the bone-regeneration field, calcium phosphate-based biomaterials (CaPs) present excellent biocompatibility, bioactivity, and biodegradability due to their chemical and structural similarity to the mineral phase of natural human bone [1,2,3]. Among the CaPs, hydroxyapatite (HA), Ca_5_(PO_4_)_3_(OH), deserves special attention since it is the main inorganic component of bone tissue (70%), with the remaining 30% being composed of organic collagen materials and bone marrow cells [4]. In addition to similar chemical properties, HA also has physical-mechanical features comparable to bone. Furthermore, the biological characteristics of HA, show osteoconductive and osteoinductive properties as verified by in vitro and in vivo studies. These properties allow bone-cell attachment, proliferation, and migration, regardless of how the HA is used (i.e., coating, powder, bulk, or porous scaffolds), thus promoting rapid new bone formation and allowing the new bone to bond to the host (osteointegration) [5,6,7,8].

Due to the disadvantages of synthetic hydroxyapatite regarding low durability and stability, hydroxyapatite of natural synthesis, through natural bio residues, has presented a good solution for treatment feasibility in bone regeneration. Furthermore, the conversion of bio-waste into useful bioproducts, which is a low-cost practice, is also a more dynamic response for the environment [5,9,10]. Thus, researchers have suggested hydroxyapatite from natural sources as a promising material for direct application in the field of regenerative medicine.

HA can be synthesized or extracted from various natural sources such as fish scales/bones, bovine bones, eggshell, or the exoskeletons of marine organisms [11]. To purify HA, heat treatment over a long time is needed to remove the organic part. However, biological HA is non-stoichiometric and it may contain certain amounts of ionic replacements and impurities [12,13]. Some of these impurities may, however, benefit bone growth and support biological functions, such as in the case of the presence of Mg^2+^, Sr^2+^, and Zn^2+^ that can increase the proliferation, bone density, and osteoblastic activity, respectively [14,15,16]

The biological properties of hydroxyapatite can be further improved by inserting some cations, including Ag^+^, Sr^2+^, Zn^2+^, Ce^3+^, Mg^2+^ [17,18]. In addition to enhancing osteoblastic proliferation, the magnesium ion is also essential for bone metabolism, promotes cell differentiation, stimulates new bone formation and mineralization, and increases bone cell adhesion [19,20,21]. In turn, its deficiency can cause a reduction in osteoclastic and osteoblastic activity, leading to bone fragility and/or reduced bone growth [19].

The bioactivity process can also be promoted through the presence of electrical charges in hydroxyapatite. Yamashita et al. found that HA can store a high density of electrical charge, which significantly increases its bioactivity level allowing much faster binding to the host-bone tissue. Osteointegration time can decrease by half compared to uncharged hydroxyapatite [21,22,23,24,25]. This process occurs because the surface loading increases osteoblast adhesion and bone mineralization in the initial phase of the bone-implant interface, through two mechanisms associated with the formation of the apatite layer (adsorbing Ca^2+^ and PO_4_^3−^), adsorbing certain types of proteins with desirable reactions with bone-forming cells [25,26].

The main objective of this work was to study naturally-synthesized hydroxyapatite as a low-cost and eco-friendly biomaterial for application in bone regeneration. Furthermore, this study evaluates the electrical behavior of the processed HA, as a potential-storing electrical-charge biomaterial, which will promote the osteointegration process.

## 2. Materials and Methods

### 2.1. Sample Preparation

**Figure 1:**
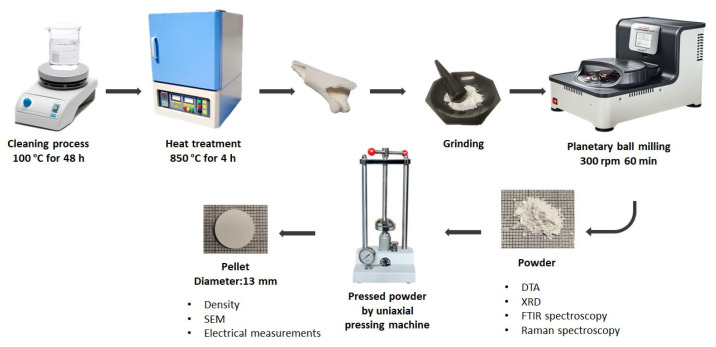
Preparation process steps.

The hydroxyapatite was obtained from sheep bones. To extract and purify the bones, the cleaning process was started by leaving the sheep bones in boiling water for two days. After, the bones were subjected to a heat treatment, in air, at 850 °C for 4 h. To obtain fine powders, the treated sheep bones were ground in an agate mortar. These powders were called sheep hydroxyapatite (sHA). In the next step, three different amounts of MgO (5 wt%, 10 wt%, and 15 wt%) were mixed with the ground sheep-bone powder. Each composition was homogenized using a ball milling planetary system operating at 400 rpm for 1 h, in dry conditions. Agate vessels and balls were used. The homogenized powder was used to prepare 13 mm diameter pellets of approximately 2 mm thickness, using a uniaxial pressure system and steel molds. For each pellet, a pressure of 2 tons was applied for 10 min. After, all pellets were subjected to a heat treatment at 600 °C, for 24 h, using a heating rate of 5 °C/min. Figure 1 represents the overall preparation process.

### 2.2. Structural and Morphological Analysis

The thermal characteristics of the composite were analyzed using differential thermal analysis (DTA) and thermogravimetric (TG) analysis. The structure of the samples was investigated through density measurements, X-ray powder diffraction (XRD), Raman, and FTIR spectroscopies.

The thermal analysis was performed using Hitachi STA7300 equipment. Each measurement ran from room temperature up to 1200 °C with a continuous nitrogen flux, and with a heating rate of 10 °C/min. Pure Al_2_O_3_ powder (99.999%) was used as the reference.

The density measurements were made using the Archimedes principle, hydrostatic weighing method, using an Adam Equipment ADP 110 (Milton Keynes, UK). Ethyl alcohol (purity > 99.5%), from Merck (Rahway, NJ, USA), was used as the liquid phase. The measurement process was repeated 10 times for each pellet to minimize measurement errors. Between measurements, each pellet was dried in a vacuum oven at 100 °C and then left to reach room temperature before a new measurement.

The XRD results were obtained at room temperature using an Aeris-Panalytical diffractometer (Malvern Panalytical, Malvern, UK). CuKα radiation (*λ* = 1.54056 Å) was used and it was working at 40 kV, and 15 mA. The scanning parameters were a scan step of 0.02°, with a time of 50 s per step and a 2*θ* angle range between 6° and 70°. From the measured data, the crystallite size was calculated by the Debye-Scherrer equation [27]:

(1) $$D=\frac{K\lambda }{\beta cos\theta }$$

where *K* was considered 0.9, *λ* is the wavelength of the X-ray beam (*λ* = 1.5418 Å for Cu Kα radiation), *β* is defined as the full width at half maximum (radian), and *θ* is the X-ray diffraction angle (°) [28]. The crystallinity degree ($X_{c}$) was also evaluated using the following equation:

(2) $$X_{c}=1-\frac{V_{112/300}}{I_{300}}$$

where $I_{300}$ is the intensity of the reflection crystal plane 300 and $V_{122/300}$ is the intensity of the deep between 112 and 300 reflections crystal plane [29].

The FTIR data was obtained using a Perkin Elmer Spectrum BX spectrometer (PerkinElmer, Waltham, MA, USA) in the range of 400–1300 cm^−1^ at room temperature. The Raman spectroscopy was performed using a Jobin Yvon HR800 spectrometer (Horiba, Kyoto, Japan) using an Ar^+^ laser (*λ* = 442 nm), a magnification lens of 50×, and the spectra were produced in a back-scattering geometry, in the range of 100–1300 cm^−1^.

The pellets morphology was evaluated using an scanning electron microscope (SEM) from TESCAN (Brno, Czech Republic), model Vega 3. Several regions of each sample were analyzed using a circular scanning area of 5 µm in diameter. All the samples’ surfaces were prior covered with carbon to enhance the surface electron conductivity. The grain sizes were measured using the ImageJ software 1.51 [30], by reading randomly the diameter of 20 grains with the boundaries well defined.

### 2.3. Electrical Analysis

In the electrical measurements, the surface of each heat-treated pellet was polished to obtain parallelism between opposite surfaces. The electrodes were made by painting the parallel surfaces with silver conducting paste (RS 186-3600 conductivity paint). A nitrogen-bath cryostat system was used to measure the electrical characteristics in the temperature range between 300 K and 400 K, controlled by an Oxford ITC 4 (Oxford Instruments, Abingdon, UK) connected to platinum temperature sensors. During the measurements, all samples were kept in a static Helium atmosphere to prevent moisture and to reduce the thermal gradients by improving the heat transfer. The ac electrical conductivity ($\sigma_{ac}$) of the samples was measured with an Agilent 4294 Network analyzer (Agilent Technologies, Santa Clara, CA, USA), working in the frequency range of 40 Hz to 1 MHz in a $C_{p}-R_{p}$ configuration [31,32]. The ac conductivity was calculated using the following equation [33,34];

(3) $$\sigma_{ac}=\varepsilon^{\prime \prime }\omega \varepsilon_{0}$$

where *ω* represents the angular frequency, $\varepsilon_{0}$ is the vacuum permittivity, and $\varepsilon^{\prime \prime }$ is the imaginary part of the complex permittivity. The real ($\varepsilon^{\prime }$) and imaginary ($\varepsilon^{\prime \prime }$) parts of the permittivity ($\varepsilon^{*}$) were calculated using Equation (4) [34,35,36,37]:

(4) $$\varepsilon^{\prime }=\varepsilon^{\prime }+j\varepsilon^{\prime \prime }=\frac{d}{S}\frac{C_{p}}{\varepsilon_{0}}+j\frac{d}{S}\frac{1}{\omega R_{p}\varepsilon_{0}}$$

where $C_{p}$ and $R_{p}$ represent the measured capacitance and resistance, respectively, *d* is the thickness of the sample, the *S* value is the electrode area, and $\varepsilon_{0}$ is the vacuum permittivity (8.854 × 10^−12^ F/m).

The activation energy ($E_{a}$) was calculated using the Arrhenius equation:

(5) $$\sigma =\sigma_{0}exp\left(-\frac{E_{a}}{K_{B}T}\right)$$

where $\sigma_{0}$ is a pre-exponential factor, $E_{a}$ is the activation energy, $K_{B}$ is the Boltzmann constant, and *T* is the temperature.

For the dc electrical conductivity ($\sigma_{dc}$) measurements, a Keithley 617 electrometer (Cleveland, OH, USA) was used, which can measure currents down to 10^−14^ A. For each measurement, it was applied a voltage (*V*) of 100 V and the current (*I*) was measured after stabilization. The conductivity was then calculated using Equation (6).

(6) $$\sigma_{dc}=\frac{Id}{VS}$$

where *d* represents the sample thickness and *S* the electrode area.

## 3. Results

**Figure 2:**
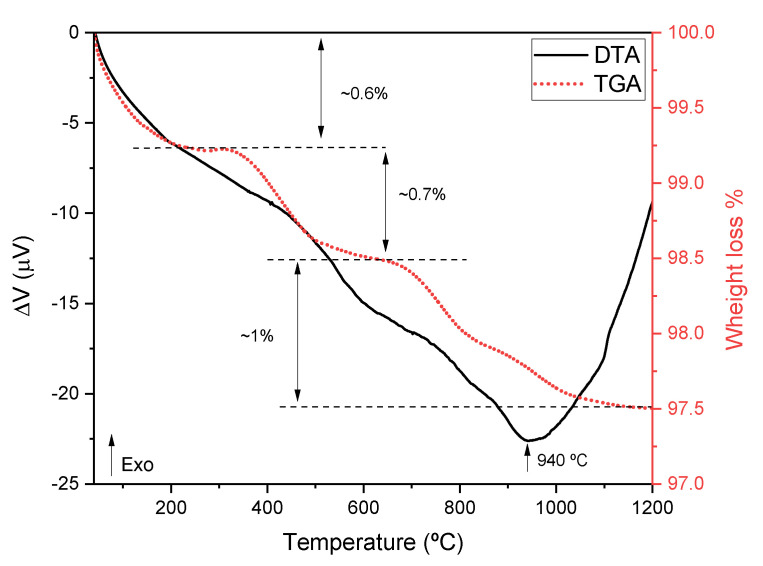
DTA and TGA spectra of sHA.

Figure 2 shows the DTA and TGA results of the sHA, showing the existence of three temperature regions with considerable mass loss. The first region, between room temperature and 270 °C, shows a mass loss of about 0.8% which should be related to the release of absorbed water and the decomposition of some residual volatile organic impurities. The second region, between 320 °C and 635 °C, reveals a mass loss of 0.7% which can be related to the release of chemically-bonded water and to the decomposition of carbonated-based impurities. The last region, above 650 °C, with a loss percentage of 1%, can be attributed to a decarbonization process, removal of residual organic moieties (i.e., collagen, fatty tissue, keratin sulphate, and chrondroitin sulphate), and to the start of the dehydroxylation processes. The endothermic phenomena centered at 940 °C can be associated with such a process [38]. Compared to the results of Sofronia et al. [39], it can be concluded that the first heat treatment at 850 °C led to the release of the major part of water and organic impurities. These results justify the choice of the temperature of 600 °C for the pellets heat treatment process because it is below the dehydroxylation starting point.

**Figure 3:**
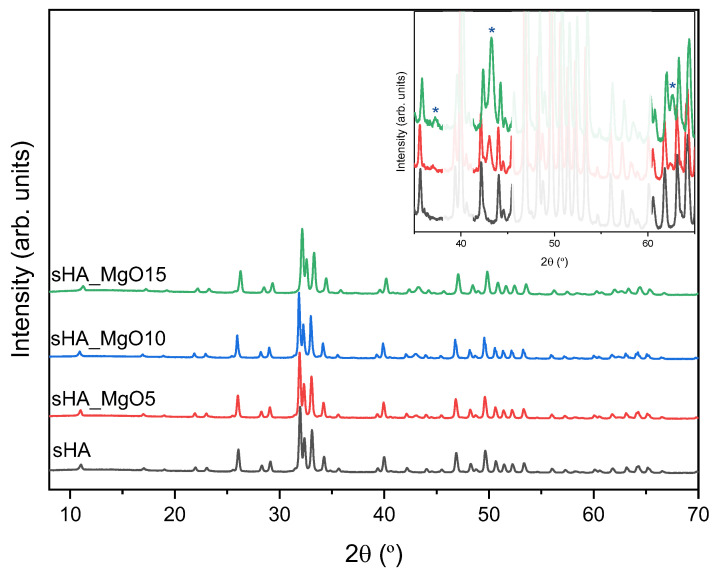
XRD results of all samples. The inset shows the samples with 5 and 15% of MgO (* are the MgO crystalline phase diffraction peaks—ICCD ref.: 04-005-4664).

The XRD pattern of pure sHA and the composites formed by sHA and MgO are shown in Figure 3. The sample sHA presents well-defined peaks associated with the Ca_5_(PO_4_)_3_(OH) crystal phase (ICCD ref. 01-080-6199) [40]. This spectrum is very similar to that obtained by Ibrain et al. [41] on hydroxyapatite from bovine, ovine, and chicken bones heat-treated at 1000 °C. The spectra of the sHA + MgO composites are similar to the sHA but, as expected, present new diffraction peaks attributed to the MgO crystalline phase (ICCD ref.: 04-005-4664), visible in the expanded spectrum (inset). With the increase of MgO content, the peaks related to the MgO phase become more evident, as visible in the inset [42,43,44]. The crystalline size of the hydroxyapatite and MgO were calculated using the Debye-Scherrer equation. Table 1 shows the obtained results. For the calculations, the same peaks were used in all samples, for the hydroxyapatite phase the 2*θ* angle used was 26.0 and for MgO 43.0. It is visible that for both phases the crystalline size is always below 100 nm, being the smallest crystalline size associated with the MgO phase.

**Figure 4:**
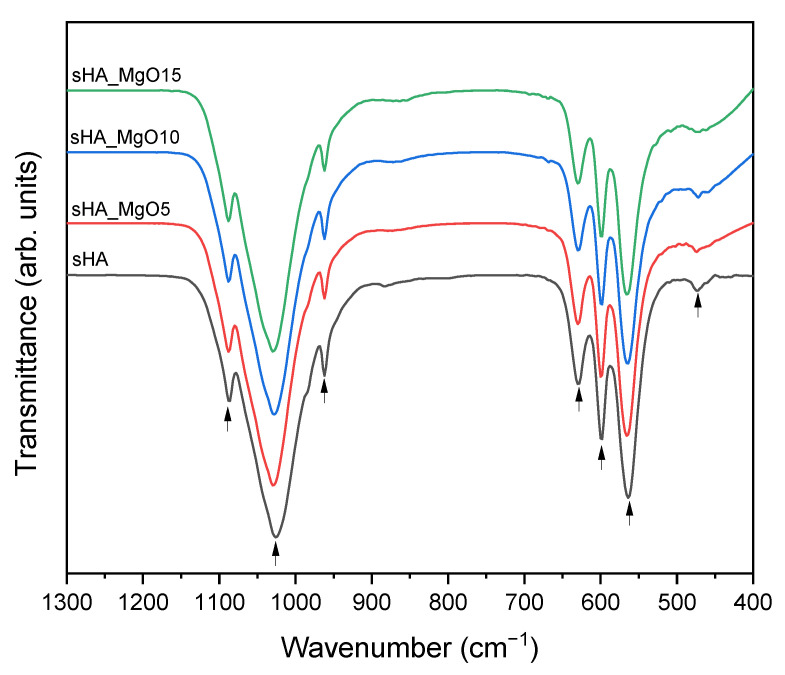
FTIR spectrum of all sHA + MgO composites and pure sHA (arrows indicate the major vibration bands).

The FTIR spectrum of all composites, shown in Figure 4, reveals a similar profile indicating that the addition of MgO does not affect the HA structure. In those spectra, the characteristic absorption bands of HA can be observed at around 474, 570, 602, 633, 962, 1051, and 1092 cm^−1^. The band centered at about 1000–1100 cm^−1^ and the intensive absorption bands around 500–600 cm^−1^ indicate the presence of PO_4_^3−^ groups. More specifically, the characteristic absorptions bands of the υ_2_ (PO_4_^3−^) and OH^−^ groups are observed at 474 cm^−1^, the υ_4_ (PO_4_^3−^) at 570 and 602 cm^−1^, υ_1_ (PO_4_^3−^) at 962 cm^−1^, and υ_3_ (PO_4_^3−^) at 1051 and 1092 cm^−1^. The band at 633 cm^−1^ is attributed to the OH- groups and can be considered proof of the presence of HA [45,46,47]. The intensity of this band does not change with the increasing amount of MgO, which indicates that Mg does not enter the HA structure [48].

**Figure 5:**
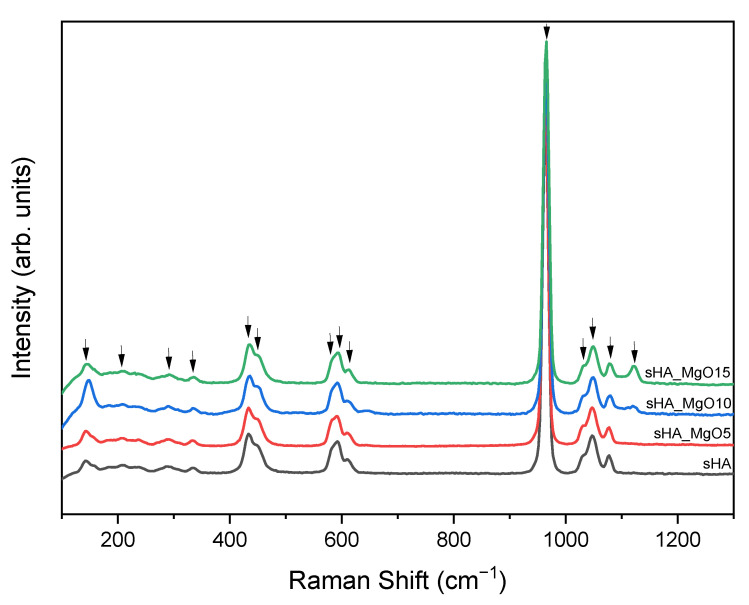
Raman spectrum of all sHA + MgO composites and pure sHA (inset is a simple magnification of the vibrations between 150 and 450 cm^−1^).

Figure 5 shows the Raman spectra of all samples and, as in the FTIR results, the different compositions reveal identical vibrations. The weak bands at 141 cm^−1^, 207 cm^−1^, and 288 cm^−1^ are assigned to the lattice mode of CaPO_4_ and the band at 332 cm^−1^ is assigned to the Ca-OH υ_3_ stretching mode. The peak at 963 cm^−1^ is related to the PO_4_^3−^ υ_1_ vibration (symmetric P-O stretching mode). The peaks at 433 cm^−1^ and 447 cm^−1^ were assigned to PO_4_^3−^ υ_2_ vibration (bending mode), and the peaks at 1030 cm^−1^, 1047 cm^−1^ and 1079 cm^−1^ corresponded to the PO_4_^3−^ υ_3_ vibrations (asymmetric P-O stretching mode). Vibrations at 582 cm^−1^, 593 cm^−1^, and 606 cm^−1^ are attributed to PO_4_^3−^ υ_4_ vibrations (asymmetric O-P-O bending mode) [46,49,50,51]. In agreement with FTIR results, the composites with higher content of MgO reveal a Raman shift at 1122 cm^−1^, attributed to a vibration associated with the MgO crystal phase [52]. This result is in agreement with the XRD spectra analysis.

**Figure 6:**
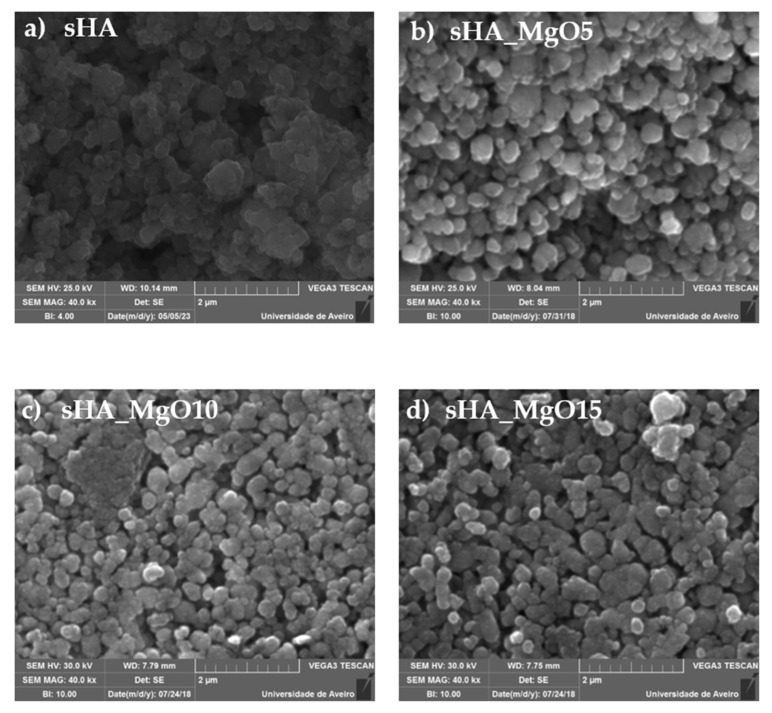
SEM micrographs of samples.

The sample morphology analysis was made using SEM microscopy and the obtained micrographs show grains with regular shapes, similar to spheres, in all samples. This habit is in agreement with that presented by Ibrahim et al. [41] in their study on hydroxyapatite from sheep, bovine, and chicken bones after heat treatment at 1000 °C. In all of our samples, different grain habits were not observed, which could be associated with the MgO phase, so it is suggested that the MgO grains must have a size much lower than the one of sHA. The micrographs of the samples with higher MgO content (Figure 6) also suggest that the addition of MgO can promote sHA grain aggregation. The sHA grain size, in all composite samples, is similar and higher than 200 nm. The pure sHA presents a grain size around 125 nm (Table 1).

The Debye-Scherrer formula applied to the XRD spectra allows calculation of the crystallite size. This characteristic was calculated for the sHA and MgO phases, and the results, shown in Table 1, revealed sizes between 50 and 80 nm for the sHA and between 17 and 19 nm for the MgO, approximately. These results revealed that the grains observed in SEM are formed by the aggregation of sHA crystallites and, as expected, the MgO should present low-size grains when compared to the sHA ones.

The density measurements show a decrease in density values with increasing MgO concentration (Table 1). Since the SEM micrographs (Figure 6) show similar profiles, including the porosity level, the observed decrease can be related to the insertion of the MgO, which mass is relative lower than that of hydroxyapatite. Comparing these results with the theoretical value for the hydroxyapatite [53], it is observed that our samples present approximately 82% of the theoretical one, revealing that the treatment conditions, mainly temperature and time, were not enough to promote higher densification.

**Figure 7:**
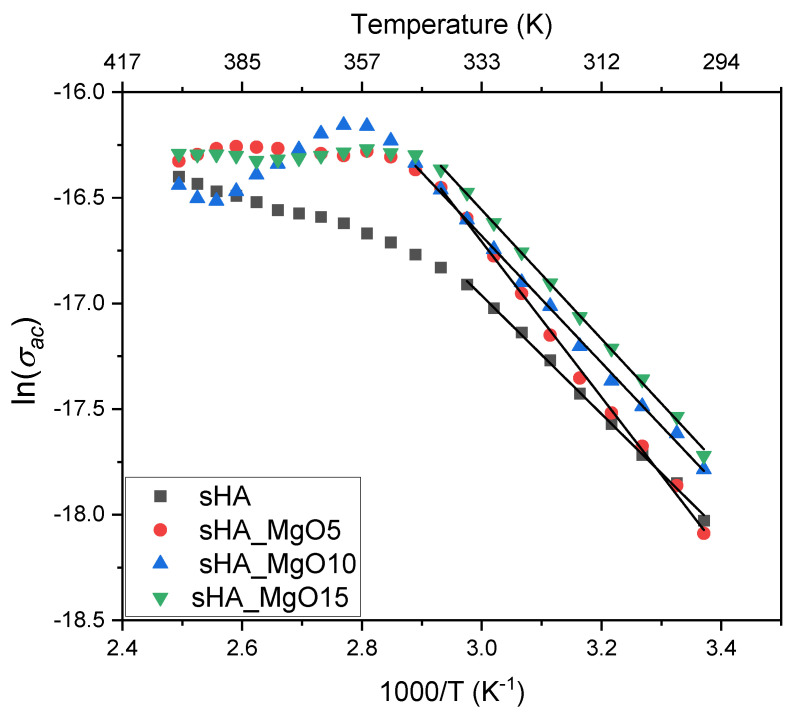
The logarithm of the ac conductivity, measured at 10 kHz, as a function of the inverse of the temperature for all samples.

Figure 7 shows the ac conductivity dependence with the measurement temperature for all samples. The presence of two regions with different profiles is visible, the first between room temperature and 340 K, where the conductivity is thermally activated, and a second region, between 350 K and 400 K approximately, where the conductivity tends to a constant trend.

The ac conduction-activation energy, calculated by linear regression of the conductivity data using the Arrhenius equation, which is represented in Figure 7 by the lines and the calculated values inserted in the legend of the figure, shows an augment with the increase of MgO, except for the sample with 5% that shows the highest value. Since the activation energy should be related to the potential barrier height that the charge carriers must overcome, the above results reveal that the sample with 5% of MgO has a particular structure that makes difficult the charge carriers’ motions, which are reflected in the low conductivity value. This can be related to the degree of crystallinity (Table 1). The calculated values show that the 5% MgO sample presents the highest crystallinity, indicating the lowest contribution of the remaining amorphous phase for the conductivity.

**Figure 8:**
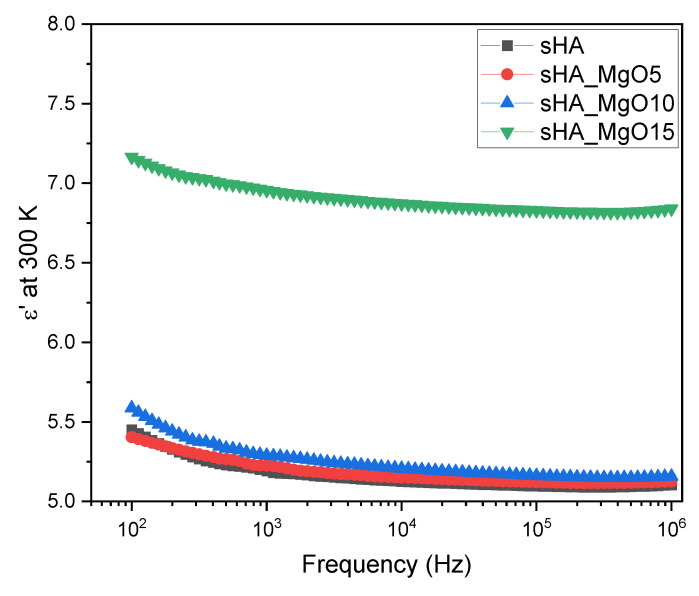
Dielectric constant as a function of the frequency of all samples.

Table 2 shows the values of the $\sigma_{ac}$, at room temperature, indicating that the increase of MgO content promotes an increase in the conductivity but it also promotes an increase in the dielectric constant. The increase in the polarization term and in the conductivity term makes the dielectric loss values $tan\delta =\frac{\varepsilon^{\prime }}{\varepsilon^{\prime \prime }}$ of the sHA and sHA_MgO15 very similar, which indicates that the sample with higher content of MgO, which shows the highest $\varepsilon^{\prime }$ value is more suitable for electrical charge storage. Moreover, these samples show an $\varepsilon^{\prime }$ quasi-frequency independent behavior (Figure 8).

## 4. Conclusions

In this study, the electrical properties of sheep-bones-derived hydroxyapatite mixed with magnesium oxide are considered and related to its structural and morphologic characteristics. The XRD revealed that the milling process favors the mixture of MgO with HA without substantially changing its structure. From the analysis of the XRD spectra it is concluded that the crystallite size of HA is larger than that of MgO and suggests that the degree of crystallinity tends to decrease for higher MgO concentrations (*X_c_* = 85.7%). Raman and FTIR spectroscopies are in agreement with the XRD results, revealing vibrations associated with the MgO crystalline phase. FTIR spectra also indicate that Mg ions do not incorporate into the HA structure (constant intensity of the band at 633 cm^−1^). At the morphological level, it is concluded that the insertion of MgO up to 15% does not promote significant alteration of the grain habit. However, SEM reveals an increase in grain size with increasing MgO concentration (125.57 nm up to 238.79 nm). SEM also shows an affinity for crystallite aggregation. The electrical measurements showed that the sample with higher MgO content, 15%, has more potential to store electrical charges (ε′ = 6.87 at 300 K and 10 kHz) and has lower relative losses (tan δ = 6.3 × 10^−3^, at the same experimental condition). It is also noted that the 5% of MgO samples show the highest crystallinity and the lowest conductivity behaviors.

## Figures and Tables

**Table 1:** The density, crystalline size (*D*), crystallinity degree (*X_c_*), and grain size of all sHA + MgO composites and pure sHA.

Sample	Density [g/cm^3^]	*D* [nm]		*X_c_* [%]	Grain Size [mm]
		sHA	MgO		
sHA	2.59 ± 0.28	58.4 ± 4.1	--	88.7	125.57 ± 28.36
sHA_MgO5	2.49 ± 0.19	65.8 ± 4.9	18.8 ± 0.4	89.5	217.35 ± 40.55
sHA_MgO10	2.42 ± 0.24	81.3 ± 6.7	17.3 ± 0.4	89.4	235.04 ± 44.05
sHA_MgO15	2.41 ± 0.28	49.9 ± 3.1	16.9 ± 0.3	85.7	238.79 ± 48.95

**Table 2:** DC conductivity at 300 K, dielectric constant and loss and the AC conductivity of all sHA_MgO composites and pure sHA.

Sample	$\sigma_{dc}$ [S/m]	${ɛ}^{\prime }$	*tan δ*	$\sigma_{ac}$ [S/m]
		[@300 K; 10 kHz]		
sHA	3.4 × 10^−10^	5.13	6.2 × 10^−3^	1.8 × 10^−8^
sHA_MgO5	2.6 × 10^−10^	5.15	6.1 × 10^−3^	1.7 × 10^−8^
sHA_MgO10	3.2 × 10^−10^	5.21	8.8 × 10^−3^	2.5 × 10^−8^
sHA_MgO15	6.1 × 10^−11^	6.87	6.3 × 10^−3^	2.4 × 10^−8^

## Data Availability

Data and materials are available under request.

## References

[1] Gavinho S.R., Pádua A.S., Sá-Nogueira I., Silva J.C., Borges J.P., Costa L.C., Graça M.P.F. (2022). Biocompatibility, Bioactivity, and Antibacterial Behaviour of Cerium-Containing Bioglass^®^. Nanomaterials.

[2] Gavinho S.R., Prezas P.R., Graça M.P.F. (2017). Synthesis, structural and electrical properties of the 45S5 Bioglass^®^. Electrical Measurements: Introduction, Concepts and Applications.

[3] Gavinho S.R., Graça M.P.F., Prezas P.R., Silva C.C., Freire F.N., Almeida A.F., Sombra A.S.B. (2019). Physical and Biological Properties of Biomaterials Intended for Bone Repair Applications. Materials Research Foundations.

[4] Gavinho S.R., Soares M.C., Borges J.P., Silva J.C., Nogueira I.S., Graça M.P.F., Petkov P., Achour M., Popov C. (2020). Preparation and Characterization of Zinc and Magnesium Doped Bioglasses. NATO Science for Peace and Security Series B: Physics and Biophysics.

[5] Sathiyavimal S., Vasantharaj S., LewisOscar F., Selvaraj R., Brindhadevi K., Pugazhendhi A. (2020). Natural organic and inorganic–hydroxyapatite biopolymer composite for biomedical applications. Prog. Org. Coat..

[6] Awasthi S., Pandey S.K., Arunan E., Srivastava C. (2021). A review on hydroxyapatite coatings for the biomedical applications: Experimental and theoretical perspectives. J. Mater. Chem. B.

[7] Raut H.K., Das R., Liu Z., Liu X., Ramakrishna S. (2020). Biocompatibility of Biomaterials for Tissue Regeneration or Replacement. Biotechnol. J..

[8] Qu H., Fu H., Han Z., Sun Y. (2019). Biomaterials for bone tissue engineering scaffolds: A review. RSC Adv..

[9] Jaber H.L., Hammood A.S., Parvin N. (2018). Synthesis and characterization of hydroxyapatite powder from natural Camelus bone. J. Aust. Ceram. Soc..

[10] Kalpana M., Nagalakshmi R. (2022). Nano Hydroxyapatite for Biomedical Applications Derived from Chemical and Natural Sources by Simple Precipitation Method. Appl. Biochem. Biotechnol..

[11] Gutiérrez-Prieto S.J., Fonseca L.F., Sequeda-Castañeda L.G., Díaz K.J., Castañeda L.Y., Leyva-Rojas J.A., Salcedo-Reyes J.C., Acosta A.P. (2019). Elaboration and Biocompatibility of an Eggshell-Derived Hydroxyapatite Material Modified with Si/PLGA for Bone Regeneration in Dentistry. Int. J. Dent..

[12] Mucalo M.R. (2015). Hydroxyapatite (HAp) for Biomedical Applications.

[13] Cursaru L.M., Iota M., Piticescu R.M., Tarnita D., Savu S.V., Savu I.D., Dumitrescu G., Popescu D., Hertzog R.G., Calin M. (2022). Hydroxyapatite from Natural Sources for Medical Applications. Materials.

[14] Ramesh S., Loo Z.Z., Tan C.Y., Chew W.J.K., Ching Y.C., Tarlochan F., Chandran H., Krishnasamy S., Bang L.T., Sarhan A.A.D. (2018). Characterization of biogenic hydroxyapatite derived from animal bones for biomedical applications. Ceram. Int..

[15] Balu S.K., Sampath V., Andra S., Alagar S., Manisha Vidyavathy S. (2021). Fabrication of carbon and silver nanomaterials incorporated hydroxyapatite nanocomposites: Enhanced biological and mechanical performances for biomedical applications. Mater. Sci. Eng. C.

[16] Gao C., Peng S., Feng P., Shuai C. (2017). Bone biomaterials and interactions with stem cells. Bone Res..

[17] Sebastiammal S., Lesly Fathima A.S., Alarifi S., Mahboob S., Henry J., Kavipriya M.R., Govindarajan M., Nicoletti M., Vaseeharan B. (2022). Synthesis and physicochemical characteristics of Ag-doped hydroxyapatite nanoparticles, and their potential biomedical applications. Environ. Res..

[18] Nandi S.K., Mahato A., Kundu B., Mukherjee P. (2016). Doped Bioactive Glass Materials in Bone Regeneration. Advanced Techniques in Bone Regeneration.

[19] Bellucci D., Sola A., Salvatori R., Anesi A., Chiarini L., Cannillo V. (2017). Role of magnesium oxide and strontium oxide as modifiers in silicate-based bioactive glasses: Effects on thermal behaviour, mechanical properties and in-vitro bioactivity. Mater. Sci. Eng. C.

[20] Coelho C.C., Padrão T., Costa L., Pinto M.T., Costa P.C., Domingues V.F., Quadros P.A., Monteiro F.J., Sousa S.R. (2020). The antibacterial and angiogenic effect of magnesium oxide in a hydroxyapatite bone substitute. Sci. Rep..

[21] Bodhak S., Bose S., Bandyopadhyay A. (2010). Electrically polarized HAp-coated Ti: In vitro bone cell-material interactions. Acta Biomater..

[22] Nakamura S., Kobayashi T., Yamashita K. (2004). Numerical osteobonding evaluation of electrically polarized hydroxyapatite ceramics. J. Biomed. Mater. Res.-Part A.

[23] Nakamura S., Kobayashi T., Nakamura M., Yamashita K. (2009). Enhanced in vivo responses of osteoblasts in electrostatically activated zones by hydroxyapatite electrets. J. Mater. Sci. Mater. Med..

[24] Yamashita K., Kitagaki K., Umegaki T. (1995). Thermal Instability and Proton Conductivity of Ceramic Hydroxyapatite at High Temperatures. J. Am. Ceram. Soc..

[25] Petrov I., Kalinkevich O., Pogorielov M., Kalinkevich A., Stanislavov A., Sklyar A., Danilchenko S., Yovcheva T. (2016). Dielectric and electric properties of new chitosan-hydroxyapatite materials for biomedical application: Dielectric spectroscopy and corona treatment. Carbohydr. Polym..

[26] Prezas P.R., Dekhtyar Y., Sorokins H., Costa M.M., Soares M.J., Graça M.P.F. (2022). Electrical charging of bioceramics by corona discharge. J. Electrostat..

[27] Silva C.C., Graça M.P.F., Valente M.A., Sombra A.S.B. (2007). Crystallite size study of nanocrystalline hydroxyapatite and ceramic system with titanium oxide obtained by dry ball milling. J. Mater. Sci..

[28] Lett J.A., Sagadevan S., Kaliaraj G.S., Alagarsamy K., Arumugam S., Sivaprakash P., Muthukumaran S., Paiman S., Mohammad F., Al-Lohedan H.A. (2020). Synthesis, characterization, and electrical properties of alkali earth metal-doped bioceramics. Mater. Chem. Phys..

[29] Chuprunov K., Yudin A., Leybo D., Lysov D., Kolesnikov E., Kuznetsov D., Godymchuk A., Mandal A.R. (2020). The Hydrothermal Synthesis Duration Influence on Calcium Phosphate and Hydroxyapatite Phase Composition. IOP Conference Series: Materials Science and Engineering.

[30] Schneider C.A., Rasband W.S., Eliceiri K.W. (2012). HISTORICAL commentary NIH Image to ImageJ: 25 years of image analysis. Nat. Methods.

[31] Graça M.P.F., Ferreira Da Silva M.G., Sombra A.S.B., Valente M.A. (2007). Electrical characterization of SiO_2_:LiNbO_3_ glass and glass-ceramics using dc conductivity, TSDC measurements and dielectric spectroscopy. J. Non-Cryst. Solids.

[32] Graça M.P.F., Ferreira da Silva M.G., Sombra A.S.B., Valente M.A. (2007). Electric and dielectric properties of a SiO_2_-Na_2_O-Nb_2_O_5_ glass subject to a controlled heat-treatment process. Phys. B Condens. Matter.

[33] Rao K.J. (2002). Structural Chemistry of Glasses.

[34] Macdonald J.R. (1992). Impedance spectroscopy. Ann. Biomed. Eng..

[35] Graça M.P.F., Ferreira Da Silva M.G., Sombra A.S.B., Valente M.A. (2006). Electrical and dielectrical properties of SiO_2_-Li_2_O-Nb_2_O_5_ glass and glass-ceramics obtained by thermoelectric treatments. J. Non-Cryst. Solids.

[36] Gavinho S.R., Graça M.P.F., Prezas P.R., Kumar J.S., Melo B.M.G., Sales A.J.M., Almeida A.F., Valente M.A. (2021). Structural, thermal, morphological and dielectric investigations on 45S5 glass and glass-ceramics. J. Non-Cryst. Solids.

[37] Gavinho S.R., Prezas P.R., Ramos D.J., Sá-Nogueira I., Borges J.P., Lança M.C., Silva J.C., Henriques C.M.R., Pires E., Kumar J.S. (2019). Nontoxic glasses: Preparation, structural, electrical and biological properties. Int. J. Appl. Ceram. Technol..

[38] Malla K.P., Regmi S., Bhattarai S., Yadav R.J., Sakurai S., Adhikari R. (2020). Extraction and Characterization of Novel Natural Hydroxyapatite Bioceramic by Thermal Decomposition of Waste Ostrich Bone. Int. J. Biomater..

[39] Sofronia A.M., Baies R., Anghel E.M., Marinescu C.A., Tanasescu S. (2014). Thermal and structural characterization of synthetic and natural nanocrystalline hydroxyapatite. Mater. Sci. Eng. C.

[40] Londoño-restrepo S.M., Jeronimo-cruz R., Millán-malo B.M., Rivera-muñoz E.M., Rodriguez-garcía M.E. (2019). Effect of the Nano Crystal Size on the X-ray Diffraction Patterns of Biogenic Hydroxyapatite from Human, Bovine, and Porcine Bones. Sci. Rep..

[41] Atemni I., Ouafi R., Hjouji K., Mehdaoui I., Ainane A., Ainane T., Taleb M., Rais Z. (2023). Extraction and characterization of natural hydroxyapatite derived from animal bones using the thermal treatment process. Emerg. Mater..

[42] Awwad N.S., Alshahrani A.M., Saleh K.A., Hamdy M.S. (2017). A novel method to improve the anticancer activity of natural-based hydroxyapatite against the liver cancer cell line HepG2 using mesoporous magnesia as a micro-carrier. Molecules.

[43] Gomes F.D.C., de Amorim J.D.P., da Silva G.S., de Souza K.C., Pinto A.F., Santos B.S., de Santana Costa A.F. (2020). Preparation and Characterization of Hydroxyapatite by the precipitation method and heat treatment. Res. Soc. Dev..

[44] Sahmani S., Saber-Samandari S., Khandan A., Aghdam M.M. (2019). Influence of MgO nanoparticles on the mechanical properties of coated hydroxyapatite nanocomposite scaffolds produced via space holder technique: Fabrication, characterization and simulation. J. Mech. Behav. Biomed. Mater..

[45] Bano N., Jikan S.S., Basri H., Adzila S., Zago D.M. (2019). XRD and FTIR study of A&B type carbonated hydroxyapatite extracted from bovine bone. AIP Conference Proceedings.

[46] Mota B., Mosquim V., Jos L., Azevedo-silva D., Aline L., Santos D., Padovini S., Geraldo A., Alberto C., Lisboa-filho P.N. (2023). Production of bovine hydroxyapatite nanoparticles as a promising biomaterial via mechanochemical and sonochemical methods. Mater. Chem. Phys..

[47] Moradi A., Pakizeh M., Ghassemi T. (2022). A review on bovine hydroxyapatite; Extraction and characterization. Biomed. Phys. Eng. Express.

[48] Manoj M., Mangalaraj D., Ponpandian N., Viswanathan C. (2015). Core-shell hydroxyapatite/Mg nanostructures: Surfactant free facile synthesis, characterization and their in vitro cell viability studies against leukaemia cancer cells (K562). RSC Adv..

[49] Viana J.R., Macêdo A.A.M., Santos A.O.D., Façanha Filho P.D.F., Graça M.P.F., Valente M.A., Silva C.C.D. (2020). Comparative analysis of solid state hydroxyapatite synthesis. Rev. Mater..

[50] Ningsih H.S., Liu Y.C., Chen J.W., Chou Y.J. (2022). Effects of strontium dopants on the in vitro bioactivity and cytotoxicity of strontium-doped spray-dried bioactive glass microspheres. J. Non-Cryst. Solids.

[51] Timchenko P.E., Timchenko E.V., Pisareva E.V., Vlasov M.Y., Volova L.T., Frolov O.O., Kalimullina A.R. (2018). Experimental studies of hydroxyapatite by Raman spectroscopy. J. Opt. Technol..

[52] Weibel A., Mesguich D., Chevallier G., Flahaut E., Laurent C. (2018). Fast and easy preparation of few-layered-graphene/magnesia powders for strong, hard and electrically conducting composites. Carbon.

[53] Prakasam M., Locs J., Salma-Ancane K., Loca D., Largeteau A., Berzina-Cimdina L. (2015). Fabrication, Properties and Applications of Dense Hydroxyapatite: A Review. J. Funct. Biomater..

